# Limited Proteolysis of Cyclooxygenase-2 Enhances Cell Proliferation

**DOI:** 10.3390/ijms21093195

**Published:** 2020-04-30

**Authors:** Esraa Saadi, Rapita Sood, Ido Dromi, Ranin Srouji, Ossama Abu Hatoum, Sharon Tal, Liza Barki-Harrington

**Affiliations:** 1 Department of Human Biology, Faculty of Life Sciences, University of Haifa, Haifa 3498838, Israel; esraa_saadi@hotmail.com (E.S.); rapitasood@gmail.com (R.S.); ido1dromi@gmail.com (I.D.); raninsr91@gmail.com (R.S.); sharonlevital@gmail.com (S.T.); 2 Department of Surgery, Ha’emek Medical Center, Afula 18101, Israel; ohatoum@gmail.com

**Keywords:** Cyclooxygenase-2 (COX-2), proteolysis, proliferation, β-catenin, cleavage

## Abstract

Accumulating evidence suggests that the cyclooxygenase-2 (COX-2) enzyme has additional catalytic-independent functions. Here we show that COX-2 appears to be cleaved in mouse and human tumors, which led us to hypothesize that COX-2 proteolysis may play a role in cell proliferation. The data presented herein show that a K598R point mutation at the carboxyl-terminus of COX-2 causes the appearance of several COX-2 immunoreactive fragments in nuclear compartments, and significantly enhances cell proliferation. In contrast, insertion of additional mutations at the border of the membrane-binding and catalytic domains of K598R COX-2 blocks fragment formation and prevents the increase in proliferation. Transcriptomic analyses show that K598R COX-2 significantly affects the expression of genes involved in RNA metabolism, and subsequent proteomics suggest that it is associated with proteins that regulate mRNA processing. We observe a similar increase in proliferation by expressing just that catalytic domain of COX-2 (ΔNT- COX-2), which is completely devoid of catalytic activity in the absence of its other domains. Moreover, we show that the ΔNT- COX-2 protein also interacts in the nucleus with β-catenin, a central regulator of gene transcription. Together these data suggest that the cleavage products of COX-2 can affect cell proliferation by mechanisms that are independent of prostaglandin synthesis.

## 1. Introduction

The cyclooxygenase-2 (COX-2) enzyme catalyzes the rate-limiting step in the conversion of arachidonic acid (AA) to prostaglandins—bioactive lipids that play central roles in cardiovascular, immunological and neurological function [1]. The COX-2 monomer is an *N*-glycosylated protein of ~72 kD that contains a signal sequence followed by three domains: a small EGF-like domain, a membrane-binding domain (MBD) consistent of four α helices, and a large globular domain that contains the catalytic site of the enzyme. In terms of its localization, COX-2 is found mostly in the endoplasmic reticulum and the contiguous nuclear envelop [2]. COX-2 is a functional homodimer (i.e., comprised of two identical monomers) and although catalysis occurs only in one of the two available sites, an intact dimer is required to maintain catalytic activity [3].

Except for several tissues where it is constitutively expressed (e.g., kidney, brain), COX-2 levels are usually low but undergo rapid upregulation in response to a wide range of inflammatory and pathological signals [4]. Many epidemiologic studies in different types of cancer and chronic inflammatory diseases showed that sustained expression of COX-2 is an untoward phenomenon that is associated with a poor prognostic outcome [5,6,7]. Furthermore, COX-2-generated prostaglandins were found to enhance proliferation via stimulation of their respective receptors and activation of downstream signaling pathways [8,9]. The combination of observations of elevated chronic COX-2 expression in many of these diseases, together with the increased understanding of the involvement of prostaglandins in proliferative signaling has suggested that inhibitors of COX-2 activity may serve as efficient chemotherapeutic agents [10]. Indeed the use of non-steroidal anti-inflammatory drugs (NSAIDs), inhibitors of enzymatic activity, has been useful in the treatment and prevention of some forms of cancer [11,12]. However, in other malignancies where COX-2 expression clearly correlates with worse prognosis [13], NSAIDs are of little value, suggesting that COX-2 may have an additional activity-independent contribution to cell proliferation.

Given the potential harm of ongoing inflammation, the large amounts of COX-2 protein that accumulate over a short period must be disposed of, rapidly and efficiently. Surprisingly, whereas many studies describe the mechanisms that control the synthesis of COX-2, much less is known about the pathways that are used to eliminate this potentially harmful protein. Naive COX-2 that has not been exposed to AA is constantly synthesized in the cell and degraded by the cellular proteasome [14,15]. However, continuous activation of the enzyme with AA, as occurs during inflammation, generates free radicals that inflict structural damage to the protein and ultimately cease its activity in a mechanism of suicide inactivation [16,17]. We have recently shown that the continued exposure to AA also leads to the de novo appearance of lower molecular weight COX-2 immunoreactive bands [18], suggesting that it may undergo limited proteolysis. COX-2 was also shown to undergo proteolysis in response to pro-inflammatory cytokines, a process that enhanced its enzymatic activity [19]. However, here we show that cleavage of COX-2 leads to the formation of COX-2 fragments that are catalytically inactive but unlike the full-length COX-2, they localize to the nucleus and enhance cell proliferation.

## 2. Results

### 2.1. COX-2 Appears in Multiple Forms in Tumors

**Figure 1:**
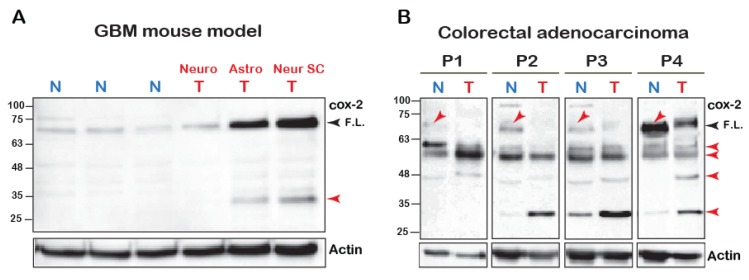
COX-2 fragments in cell lines and human samples. (**A**) 100 μg of total tissue homogenates of brain samples of glioblastoma multiforme (BGM) models of transgenic mice were resolved by SDS-PAGE and probed for the presence of COX-2. (Neuro-tumor from neuronal origin, Astro- from astrocyte origin and Neuro SC-from neural stem cells). Black arrowhead marks the full-length (F.L.) COX-2 and the red arrowheads mark the lower fragments. (**B**) Samples of tumors (T) and surrounding seemingly healthy tissues (N = Normal) obtained from four patients diagnosed with colorectal adenocarcinoma were probed for COX-2 expression. Blots show the presence of several immunoreactive bands, some of which are lower than the expected MW of the native protein (Red arrows).

The mature *N*-glycosylated form of the COX-2 monomer is reported to be ~72 kD [1]. However, we have recently reported that cleavage of COX-2 is observed upon its stimulation with AA [18]. To test whether COX-2 appears to be cleaved in vivo, we tested its expression using an antibody directed at the C-terminus of the protein (amino acids 580–599) in a mouse model of malignant glioma [20] and in tumors of patients diagnosed with colorectal adenocarcinoma. As depicted in Figure 1A, normal brain tissue of mice expresses low levels of native COX-2. In contrast, native COX-2 levels were significantly elevated in tumors originating from astrocytes and neuronal stem cells but not mature neuronal cells [20]. Interestingly, however, these samples also showed a marked expression of a distinct lower MW band at ~30 kD, which was similar to what we previously observed upon stimulation of COX-2 with AA [18].

In contrast to the mouse tumors, COX-2 expression profiles in human tumors were more heterogeneous. However, there were several similarities between all patients (Figure 1B). The most surprising finding was that in comparison to tissue from normal-seeming areas, the levels of the full-length COX-2 were significantly reduced in the tumors of all four patients. Samples from all patients also showed a relatively weak band between 63–48 kD, and three out of the four samples showed a marked increase in the band between 32–25 kD. Despite loading the same amount of total protein, actin levels were lower than the normal surrounding tissue, which highlights these differences even further. Together the data presented above indicated the presence of COX-2 immunoreactive species that are of lower molecular weight than the expected full-length form.

### 2.2. A Point Mutation at the Carboxyl-Tail Causes the Appearance of COX-2 Fragments

**Figure 2:**
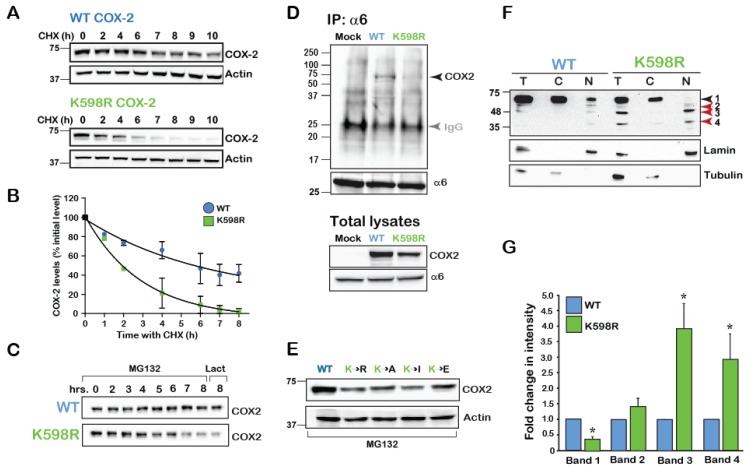
K598 COX-2 presents a fragmented pattern. (**A**) A representative immunoblot of HEK 293 cells transfected with 0.5 µg of either WT or K598R COX-2 for 16 h. Degradation of the native form (~72 kD) was studied following application of 50 µM of cycloheximide (CHX) for the indicated times. (**B**) Summary graph of *n* = 3 independent experiments depicting the decay in the levels of full-length WT and K598R COX-2. Quantification was done by comparing the levels of COX-2 over time as percent of to its initial levels at time zero. (**C**) Cells expressing either WT or K598R COX-2 were subject to proteasome inhibitors MG-132 (2 μM, 0–8 h) or lactacystin for (2 μM, 8 h). While the WT protein accumulates in response to inhibition of the proteasome, this treatment does not inhibit the reduction observed in the levels of the native form of K598R COX-2. (**D**) The proteasome 26S subunit α6 was immunoprecipitated from HEK 293 cells expressing either Mock, WT or K598R COX-2. A representative blot of n=3 depicting a unique COX-2 immunoreactive band of ~72 kD which appears only in the sample expressing WT COX-2. Probing of the total cell lysates (lower panel), confers that K598R is expressed in the cells but does not associate with α6. IgG (Grey arrow) marks the IP antibody, which appears in all three samples. (**E**) Substitution of K598 COX-2 into several amino acids with different charges yield similar phenotypes to that of K598R COX-2. 40 μg of total cell lysates of samples expressing the different COX-2 K598 mutants or WT protein were probed with anti- COX-2, 18 h after transfection. Compared to the WT protein all mutants showed significantly lower expression levels. (**F**) Representative immunoblot of samples from HEK 293 cells transfected with WT or K598R COX-2. Shown are total lysates (T) cytosolic (C) and nuclear (N) fractions, as confirmed by α-tubulin and lamin staining. Lower bands 3 and 4 (63–48 and 35–25kD, respectively) of the K598R COX-2 mutant localize in the nuclear fraction. (**G**) Summary graph showing a significant increase in the two lower bands of the K598R mutant (*n* = 8, * *p* < 0.001 vs. WT band).

To begin exploring the possibility that COX-2 may be cleaved at specific sites, and since we did not know which signals cause its cleavage endogenously, we searched for a COX-2 mutant that presents with a similar phenotype. Previous reports have indicated that in the absence of its main substrate AA, COX-2 is degraded by the cellular proteasome in a mechanism that involves a unique 19-amino acid sequence at its carboxyl terminal (C-tail) [21]. This sequence, which is conserved in vertebrates (Supplemental Figure S1), contains a single lysine in position 598 of human COX-2. Since ubiquitination of lysine residues is the first step towards proteasomal degradation, we reasoned that replacement of K598 with the positively charged arginine (R) would significantly retard COX-2 degradation by the proteasome. To test the rate of wild type (WT) and K598R mutant protein degradation we expressed both constructs in the heterologous system of HEK 293 cells, blocked de novo protein synthesis 16 h after transfection, and followed the levels of COX-2 expression over the next 10 h. To our surprise, compared to the WT COX-2, which showed a half-life of ~ 5 h, K598R COX-2 presented a much faster decay of the full-length form (t_1/2_ ~2 h, Figure 2A,B). Furthermore, inhibition of proteasomal activity by two different reagents (MG132 and lactacystin) did not attenuate the rate of decay (Figure 2C). Moreover, while WT COX-2 was found to be in association with the proteasome, K598R COX-2 was not (Figure 2D). Substitution of K598 into several amino acids with different charges and hydrophobicity consistently showed considerably less immunoreactivity compared to the WT, which was not reversed by proteasomal inhibition (Figure 2E). Together these findings indicated that the reduction in the levels of the full-length COX-2 were not due to its accelerated degradation in the proteasome.

Expression of COX-2 in HEK cells typically yields a dominant band of ~72kD, which marks the presence of the mature full-length COX-2 monomer. However, staining of total cell lysates of WT and K598R COX-2 transfected cell with anti-COX-2 against the C-terminus of the protein revealed the presence of several lower MW immunoreactive bands in the K598R COX-2 samples (Figure 2F). Enrichment of cytosolic (C) and nuclear (N) fragments of cells expressing WT or mutant COX-2 revealed the presence of the same four main immunoreactive bands. The first band (Band 1), which was visualized at ~72 kD, represents the mature *N*-glycosylated protein and was the most prominent in the WT samples, while hardly apparent in the nuclear K598R COX-2 sample. The next band (Band 2), which appeared at ~63 kD and may represent the unglycosylated form of the protein, was found in similar levels between WT and mutant. The next two bands (Bands 3 and 4) appeared at 63–48 and 35–25 kD, respectively, and were considerably elevated in the nuclear fractions of K598R COX-2 (Figure 2F,G).

**Figure 3:**
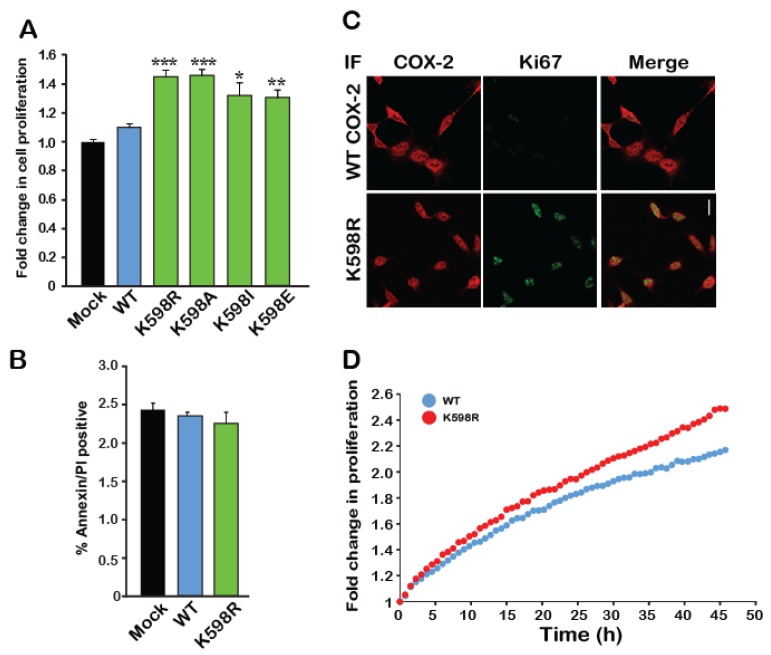
A mutation in K598 COX-2 enhances cell proliferation. (**A**) The effect of WT COX-2 and the different K598 COX-2 mutants on cell proliferation was assessed by the XTT proliferation assay, 36 h after transfection (*n* = 5 in triplicates, One-way ANOVA * *p* < 0.001 vs. mock transfection). (**B**) Expression of either WT or K598R COX-2 had no effect on the levels of annexin V or PI-positive cells (*n* = 3, in triplicates). (**C**) Co-staining of Mock, WT and K598R COX-2 with anti-hCOX-2 antibody (Red) and Ki67 (Green) shows increased localization of K598A to the nucleus and a marked increase in cell number. Scale bar represents 50 µm. (**D**) Growth of HEK 293 cells expressing either WT or K598R COX-2 was traced over 48 h using IncuCyte. Shown is the average of *n* = 3 for each condition.

Since the immunoreactive pattern of bands 3 and 4 of K598R COX-2 was similar to that which we previously observed for endogenous COX-2 expression, we tested whether the presence of K598R COX-2 in cells affects their rate of growth. We transfected an equal number of HEK 293 cells with either WT or mutant COX-2 and used an XTT proliferation assay to measure the effect on rate of proliferation. As depicted in Figure 3A, 48 h after transfection, cells expressing WT COX-2 showed similar growth rates to those transfected with empty vector (Mock). In contrast, cells expressing K598R COX-2 showed a ~1.5-fold increase in proliferation compared to the WT, as did all cells expressing different K598 substitutions. To test whether the differences in growth may be due to reduced cell death, we used flow cytometry to measure the levels of annexin V, a marker of apoptotic cells, in cells expressing WT and mutant COX-2. As shown in Figure 3B, no differences were observed in annexin V levels between the different samples concerning suggesting that the observed effect was not due to reduced apoptosis, but rather to an increase in the number of viable cells. This finding was further corroborated by staining the WT- and K598R-expressing cells with the cellular proliferation marker Ki67 (Figure 3C), which was only detectable in the nuclei of K598R-expressing cells (Figure 3C, red staining), and by live cell tracking of cell growth (Figure 3D).

Taken together, the above data suggests that mutations of COX-2 at residue K598 yield a similar immunoreactive pattern of lower form of the protein compared to the COX-2 endogenous expression in cell lines and tissues (Figure 1A,B), and that its expression enhances cell proliferation. We therefore used this construct to investigate whether the appearance of COX-2 fragments is directly connected to cell proliferation.

### 2.3. The Catalytic Domain of COX-2 Enhances Proliferation in an Activity-Independent Manner

**Figure 4:**
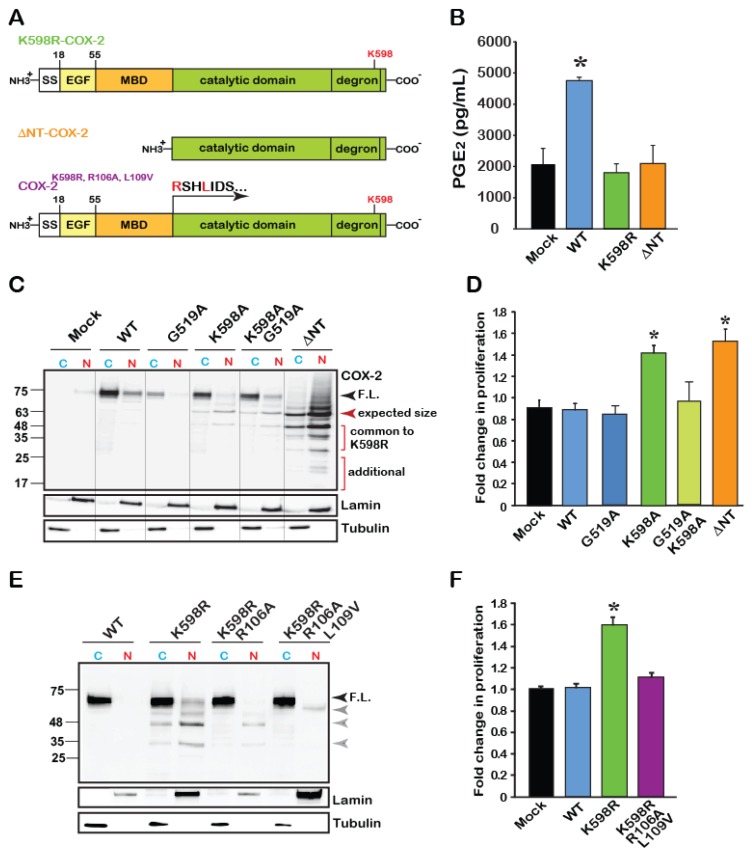
The catalytic region of COX-2 enhances cell proliferation independent of enzymatic activity. (**A**) Schematic domain structure of COX-2 and its various mutants. According to the crystal structure of COX-2, the catalytic domain begins at amino acid 110. (**B**) Measurements of WT and mutant COX-2 enzymatic activity by assessing the production of PGE_2_. Only WT COX-2 has enzymatic activity, while K598R- and ΔNT-COX-2 do not. (**C**) Representative immunoblot of Cytosolic (C) and Nuclear (N) fractions of HEK 293 cells expressing Mock transfected, WT, catalytically inactive G519A COX-2, K598R, K598A/G519 and ΔNT-COX-2. F.L. marks the full-length protein of 72 kD. COX-2 mutants contacting K598R mutations present lower MW immunoreactive bands that are mainly localized to the nucleus. A similar pattern is observed with ΔNT-COX-2, which seems to be further cleaved. (**D**) Effect of COX-2 mutants G519A, K598, G519A/K598A, and ΔNT-COX-2 on cell proliferation measured by XTT (*n* = 5 in triplicates, One-way ANOVA * *p* < 0.001vs. mock transfection). (**E**) Representative immunoblot of cytosolic (C) and nuclear (N) fractions of cells expressing WT, K598R, K598R/R106A or K598R/R106A/L109V COX-2. Mutations at the MBD-catalytic domain of K598R COX-2 cause a significant reduction in the appearance of lower COX-2 immunoreactive bands. (**F**) Mutations at the MBD-catalytic domain interface abolish the proliferative effect of K598R COX-2 as measured by XTT, 36 h post-transfection (*n* = 4 in triplicates, One-way ANOVA * *p* < 0.001vs. mock transfection).

As noted in Figure 2G, the levels of two bands, appearing around 63–48 and 25–35 kD (Bands 3 and 4, respectively) were markedly increased in the nuclear fractions of K598R samples. Since the antibody used to detect COX-2 is designed against to its C-terminus, Band 3 is roughly calculated to match the expected size of the catalytic domain of COX-2, without its EGF and membrane binding (MBD) domains (Figure 4A). We therefore tested whether the catalytic domain of COX-2 by itself, can induce cell proliferation. For this, we designed a COX-2 mutant, which lacks the N-terminal domain of the protein (without EGF and MBD, termed ΔNT-COX-2 herein) and tested its enzymatic activity, expression pattern and effect on proliferation. COX-2 activity was previously reported to require an intact homodimer, which occurs via the EGF domain of the protein. Therefore, as expected, ΔNT-COX-2 showed no enzymatic activity when exposed to AA (Figure 4B). Surprisingly, K598R COX-2 was also unable to produce PGE_2_, suggesting that site-specific cleavage may be involved in cessation of COX-2 enzymatic activity (Figure 4B).

To assess the role of catalytic activity on the pattern of COX-2 expression, we obtained cytosolic and nuclear fractions of cells expressing a catalytically inactive COX-2 (G519A-COX-2), alone or in combination with K598A-COX-2 (G519A/K598A COX-2), as well as the ΔNT-COX-2 mutant. As shown in a representative blot in Figure 4C, the catalytically inactive G519A COX-2, which binds arachidonic acid but cannot catalyze it [3,22], showed a similar expression pattern to that of the WT protein and was present mainly in the cytosolic fraction. The G519A/K598A COX-2 double mutant showed a similar pattern to that of K598A-COX-2, with the full-length form appearing in the cytosolic fraction, while the lower forms (63–48 and 35–25 kD) localized to the nuclear ones. Unexpectedly, ΔNT-COX-2 yielded a pattern of multiple distinct bands. The top band corresponded to its expected size (Figure 4C, red arrowhead); however, the next two bands showed identical migration to those observed in K598R samples, with marked nuclear localization. Of interest is also the appearance of two additional lower fragments that were unique to ΔNT-COX-2, as well as a smear above the top band that may indicate a post-translational modification such as ubiquitination (Figure 4C). These data suggest that ΔNT-COX-2 may undergo additional proteolysis following its expression. Importantly, expression of ΔNT-COX-2 induced a similar increase in cell proliferation to that of K598R COX-2 (Figure 4D) suggesting that a COX-2 protein fragment that is devoid of enzymatic activity facilitates cell proliferation. However, the active binding site must be intact, since insertion of a G519A mutant onto K598A prevents the increase in proliferation.

To further support the notion that releasing the catalytic domain may be responsible for its proliferative effect, we took an opposite approach, and inserted additional mutations at the border of the MBD-catalytic domain of the spontaneously cleaved K598R (Figure 4A, last scheme). The logic behind these mutations was that if indeed the globular domain is cleaved close to the MBD, changing the amino acids in that area will prevent the appearance of the lower bands typically seen in the K598R mutant. Probing of cytosolic and nuclear fractions of cells expressing these mutants showed a significant reduction in the presence of the K598R-typical lower bands in the nuclear fractions (Figure 4E). Simultaneous measurements of the effects of these mutants on proliferation confirmed that in the absence of the lower species of COX-2 augmentation in proliferation rate is blocked (Figure 4F).

### 2.4. K598R COX-2 Affects RNA Metabolism

**Figure 5:**
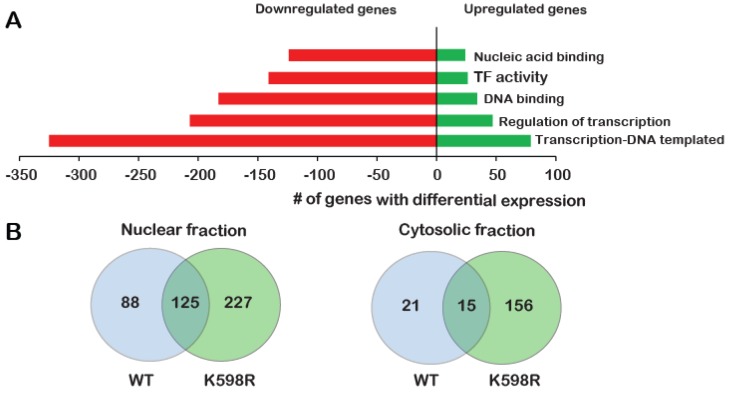

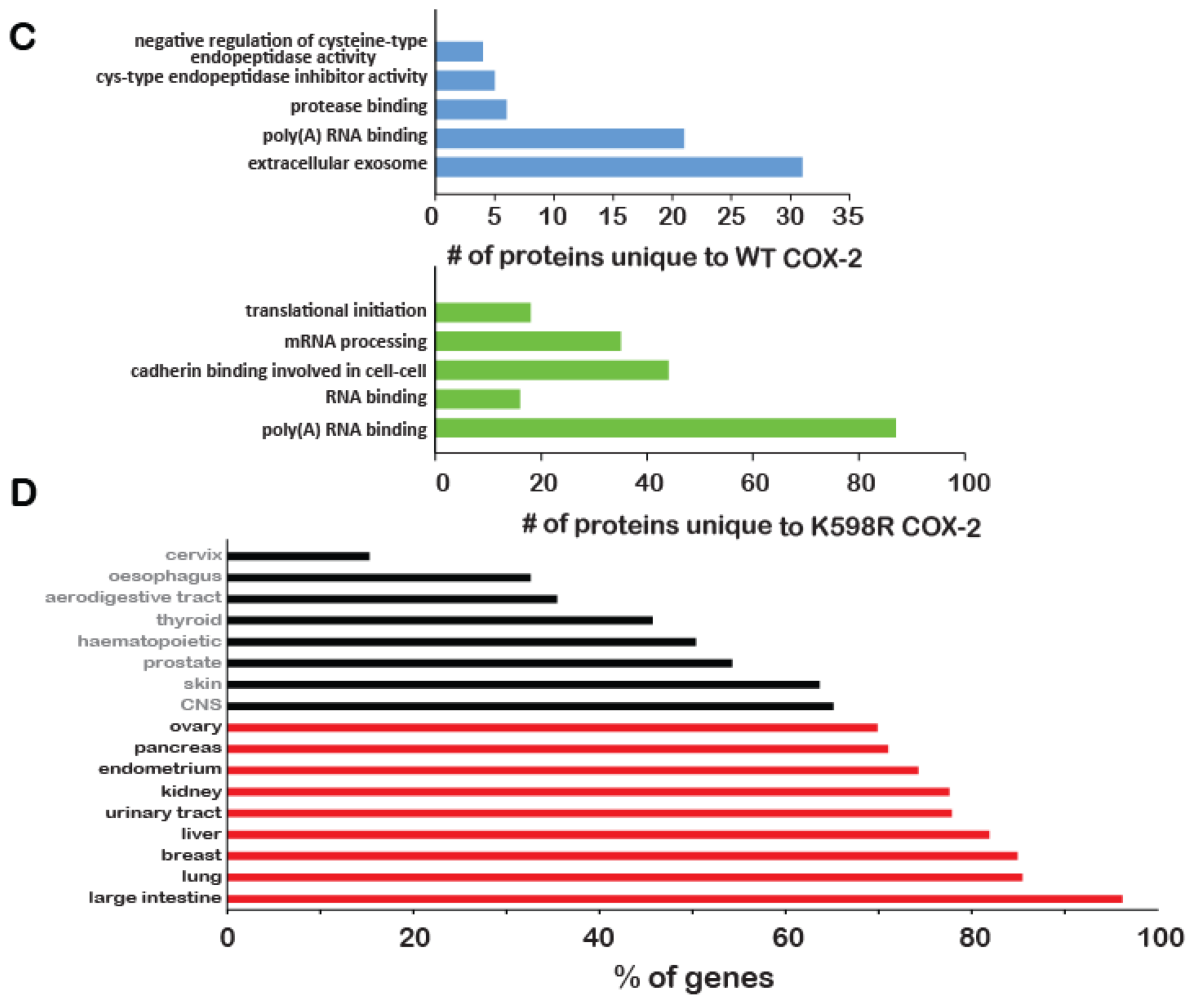
Effect of K598R COX-2 on RNA and protein expression. HEK 293 cells expressing either Mock, WT or K598R COX-2 were subject to transcriptome and proteome analyses. (**A**) Graph depicting GO enrichment analysis of K598R compared to WT COX-2 (*n* = 3 replicas of each transfection). Presented GO terms were significantly enriched (BH adjusted *p*-value <0.05) in both upregulated and downregulated subsets of differentially expressed genes. (**B**) Venn diagrams of proteins interacting with WT, K598R or both in cytosolic and nuclear fragments (*n* = 3 replicas of each transfection). (**C**) Graph depicting the GO terms of proteins that interact specifically with WT (Top) or K598R (Bottom) COX-2. (**D**) Graph depicting the percent of genes identified in the transcriptomics data as uniquely regulated in the K598R COX-2 samples involved in different types of cancer.

To identify the proliferative pathways that may be affected by K598R COX-2, we performed a transcriptome analysis of cells expressing either vector (Mock) cDNA, WT or K598R COX-2 in three biological repeats. After removing genes that appeared in the Mock lists, we compared the genes that were altered in WT vs. K598R samples. Of 63,650 genes, 461 genes showed a significant change of 2-fold or higher in expression; 80% of these genes (371) were reduced compared to the WT, while 20% (90) were elevated (Table S1). Remarkably, GO enrichment analysis showed that most of these genes are related to transcription-regulation (Figure 5A).

To further understand the unique role of cleaved COX-2 in cell proliferation, we sought to identify the cohort of proteins it interacts with. For this, we immunoprecipitated WT or K598R COX-2 from cytosolic and nuclear samples and performed a proteomic analysis to identify associated proteins (Table S2). As shown in Figure 5B, the cytosolic fractions yielded 192 proteins, while the nuclear fraction yielded more than two-fold (440). Fifteen and 125 proteins were common to WT and mutant COX-2 in the cytosolic and nuclear fractions, respectively. In both fractions, there was a significantly higher number of proteins that were unique to K598R COX-2 (156 in the cytosolic and 227 in the nuclear fraction). GO enrichment analysis of WT COX-2 unique proteins showed enrichment mainly in proteins related to extracellular exosome (Figure 5C, top panel), while K598R COX-2 showed significant enrichment of proteins involved in mRNA processing (Figure 5C, lower panel).

Given its proliferative effect, we also analyzed the representation of K598R COX-2-specific genes in different types of cancer using the Catalog of Somatic Mutations in Cancer (COSMIC, [23]). This analysis revealed enhanced prevalence of these genes in tumors of the large intestine (< 98%), followed by lung and breast cancers (Figure 5D).

### 2.5. COX-2 Interacts with β-Catenin through its Catalytic Domain

**Figure 6:**
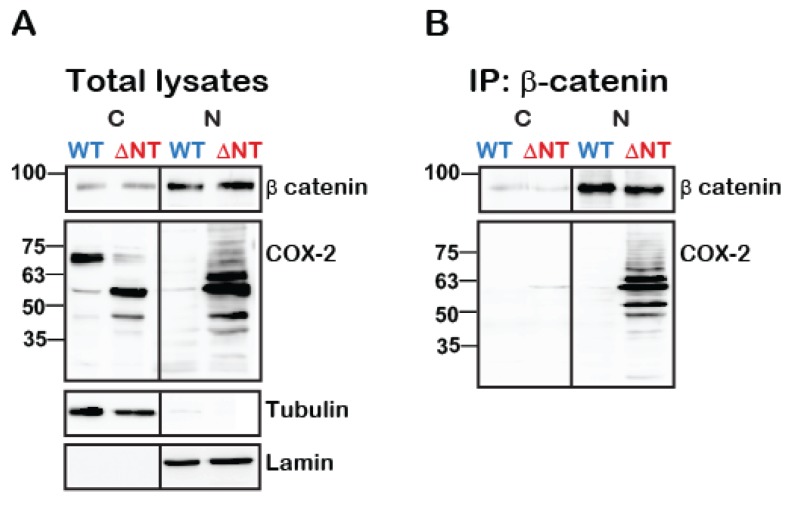
Truncated COX-2 interacts with β-catenin in the nucleus. HEK 293 cells with endogenous expression of β-catenin were transfected with either WT or ΔNT- COX-2 for 18 h. On the following day, cells were lysed, fractionated into cytosolic and nuclear fractions protein was determined and equal protein amounts underwent immunoprecipitation with anti-β catenin for 24 h at 4 °C. Cytosolic (C) and nuclear (N) fractions are confirmed by tubulin and lamin staining, respectively. (**A**) Total lysate separation shows that WT and ΔNT- COX-2 are equally expressed as is endogenously expressed β-catenin. (**B**) COX-2 is only present in the nucleic fractions of ΔNT but not WT COX-2 (*n* = 3 independent experiments).

Given the data indicating a role for cleaved COX-2 in colon cancer (Figure 1B, Figure 5D), we next tested whether WT or truncated COX-2 interact with one of the main multi-functional regulators of gene transcription: β-catenin. At resting conditions, β-catenin forms a complex with E-cadherins at the plasma membrane and with Axin and adenomatous polyposis coli (APC) in the cytosol. Activation of the Wnt pathway releases β-catenin from the APC/Axin destruction complex and promotes its nuclear translocation and association with T-cell factor/lymphoid enhancer factor (TCF/LEF) to regulate transcription [24]. β-catenin also stabilizes COX-2 mRNA by binding to its 3′-untranslated region (3′UTR) [25]. To determine whether β-catenin and COX-2 also interact with each other at the protein level we expressed WT or ΔNT COX-2 in HEK 293 cells, immunoprecipitated β-catenin from cytosolic and nuclear fractions and probed the samples for the presence of COX-2. Examination of the total cell lysates indicated that total β-catenin levels are not affected by the presence of WT or ΔNT COX-2 (Figure 6A, top panel). Furthermore, we found that both WT and ΔNT COX-2 are expressed to a similar degree, but that their cellular location is different. WT COX-2 is mostly cytosolic, while ΔNT COX-2 is localized primarily in the nuclear functions. However, when we examined the immunoprecipitates, we discovered that there is a significant interaction between β-catenin and ΔNT COX-2 in the nuclear fractions (Figure 6B).

## 3. Discussion

Due to its irreversible nature, unlike other post-translational modifications (e.g., phosphorylation, glycosylation), proteolysis is a powerful means of either activating or arresting protein function. Endogenously expressed COX-2 has previously been shown to undergo limited proteolysis in response to several pro-inflammatory factors such as TNFα IL-17 and PMA [19], an event that was found to augment the catalytic activity of the protein. We were unable to obtain COX-2 fragments using these reagents, possibly due to a different experimental system and types of antibody used. We therefore chose to study the cellular role of COX-2 proteolysis using a COX-2 mutant that undergoes spontaneous cleavage, in order to provide a proof of principle that COX-2 fragments that lack enzymatic activity can have a profound effect on cell proliferation. While the endogenous signals that may produce a similar effect are still unknown, our data highlight an important role for the degradation of COX-2 in its function. The lysine in position K598 is located at the C-tail of the protein, within a special 19-amino acid sequence that destabilizes COX-2 compared to COX-1 isoform [21]. Interestingly, the crystal structure of COX-2 does not include the amino acids in this region, suggesting perhaps that it is an intrinsically disordered area. Given our observations that K598R COX-2 yields cleavage products of fixed molecular weights, it is possible that interactions of this lysine with other parts of the protein (e.g., the MBD-catalytic domain border), serve to protect the protein from spontaneous cleavage. While the identity of the proteases that cleave COX-2 is still under investigation, our results, together with those of Mancini et al., suggest that depending on the cellular conditions and its conformation, COX-2 is may be subject to cleavage by more than one protease, with different end results.

Why is cleavage of COX-2 largely overlooked? Examination of the vast body of literature regarding COX-2 protein expression in a multitude of human tissues and diseases reveals the use of two principle detection techniques: immunohistochemistry and Western blotting. While immunohistochemistry is an excellent technique to determine protein expression and localization, it does not provide critical information regarding the actual size of the protein. This is particularly the case if the antibody used in the experiment is directed against parts of the protein that remain intact after proteolysis. In studies that take the Western blot approach, images seldom present the full gel, but rather tend to portray only the portion that corresponds to the full-length protein. In their study of COX-2 proteolysis in human fibroblasts, Mancini et al. used an anti-pan-human COX-2 polyclonal antibody to identify COX-2 immunoreactive bands of 72, 66, 54, 34–36 and 28 kD [19]. In the current study, we used antibodies directed against the C-tail of COX-2, and estimated the size of cleavage products based on their migration in the 1D-gel. Our data is similar in most cases to that of Manchini et al., with the most prominent bands appearing at 72, 66–63, 52–54, and 32 kD (Figure 2F,G). Whereas the epitope of the antibody used in that study is not mentioned, we assume it is directed against the C-tail of the protein. We have also tried to use antibodies against the N-terminus of the protein but unfortunately, none were of sufficient quality to provide the complementary information.

The use of common proteomics for identifying protein cleavage sites is also problematic due to the inability of common proteomic procedures to distinguish between naturally occurring fragments and those that are artificially generated by sample preparation for mass spectrometry. Often these methods include subjecting the samples to tryptic cleavage by proteases such as trypsin or chymotrypsin, which cleave proteins mainly on the carboxyl side of lysine or arginine. The report by Mancini et al. regarding COX-2 cleavage identified seven tryptic sequences in native COX-2 using liquid chromatography tandem mass spectrometry [19]. Six of these fragments belonged to the catalytic domain and all contain a lysine or arginine at their C-terminus, making it impossible to determine whether they are true in vivo proteolytic products. The seventh product, which is the only one that does not end with a lysine or arginine (RSHLIDSPPTYNADYGY), corresponds to the border of the MBD-catalytic domain and is similar to our prediction that one of the fragments observed in K598R COX-2 occurs in this region (Figure 4). We further show that separation of the catalytic domain from its membrane binding elements (ΔNT-COX-2) results in a protein that undergoes nuclear translocation and affects proliferation, and that mutations in the MBD-catalytic domain area prevent these effects (Figure 4E,F). Identification of the true COX-2 proteolytic products requires the use of more complex mass spectrometry methods that enrich or label the N-termini of naturally occurring peptides in order to separate them from peptides form during sample preparation [26,27].

One of the surprising findings of the study was the profound effect of truncated COX-2 on RNA metabolism. Furthermore, more than 40 of the proteins that were associated only with the truncated protein were E-cadherin binding proteins (Figure 5C), and the majority of the genes that changed significantly in the K598R COX-2 samples were overrepresented in colon cancer (Figure 5D). Common to all three aspects is β-catenin, a multi-functional protein that is indicated in over 90% of all cases of sporadic colon cancers [28]. The classical mitogenic effect of β-catenin is through its translocation to its nucleus upon release from the Axin/APC destruction complex, where it binds to the 5′UTR of the transcription factor TCF/LEF and drives the expression of mitogenic target oncogenes such as *cyclin D1* and *c-MYC* [29,30]. However, β-catenin also regulates RNA metabolism [31], binds to 3′UTR regions of COX-2 mRNA via its Armadillo (Arm) domain [25], and interacts with different RNA stabilizing proteins [32]. Therefore, the oncogenic effect of β-catenin is mediated by affecting gene expression at multiple levels. In the present study, we used constructs that contain only the coding regions of COX-2 mRNA; therefore, the observed effect of truncated COX-2 on proliferation is not mediated by regulating mRNA stability. Furthermore, although prostaglandins, the products of COX-2 catalysis, were shown to activate the Wnt/β-catenin pathway [33], it is unlikely that the observed effects of truncated COX-2 on proliferation is mediated by them, since the only interaction is observed with ΔNT-COX-2, which lacks catalytic activity (Figure 6B). Instead, we propose the existence of an additional level of feedback into the action of β-catenin, through protein interaction with the catalytic region of COX-2.

Overexpression of COX-2 in colorectal cancer and its association with poor prognosis are well documented. Inhibition of COX-2 activity by NSAIDs is advantageous in delaying the onset of the disease among patients with familial adenomatos polyposis (FAP) who develop colon cancer in early adulthood due to the loss of ability to inhibit the Wnt/β-catenin pathway [34], whose polyps contain high levels of COX-2 [35]. However, given the biology of COX-2 function, the presence of high inflammation within the tumors may cause continuous activation of the enzyme, which leads to suicide inactivation. This in turn can culminate in cleavage of COX-2 and the generation of fragments that upregulate cell proliferation in a prostaglandin-independent manner. Support for this idea comes from a recent study that examined the level of COX-2 activity within human colon tumors and found that although COX-2 levels were elevated, its activity was significantly lower than in healthy areas. Interestingly, among the most prominent proteins within tumor samples, almost 30% were linked to RNA splicing [36], which may affect the general transcriptional program of the cell.

In conclusion, the data presented herein supports a role for COX-2 fragments in augmenting cell proliferation via a mechanism that does not involve classical prostaglandin receptor signaling. Identification of the protease that cleave COX-2 and their respective cleavage sites is under investigation and may provide additional new means to treat proliferative and chronic inflammatory diseases.

## 4. Materials and Methods

### 4.1. Materials

(s)-MG132 and arachidonic acid (AA) were purchased from Cayman Chemical (Ann Arbor, MI, USA), cycloheximide and lactacystin were from Sigma Aldrich (Saint Louis, MI, USA). All cell culture media, fetal bovine serum and antibiotics were from Biological Industries (Beit HaEmek, Israel). All other materials were standard laboratory grade.

### 4.2. Antibodies

Goat polyclonal anti-COX-2 (human) (C-20, sc-1745), mouse polyclonal anti-Lamin B (human) (M-20, sc-6217), mouse monoclonal anti-alpha Tubulin (human) (A-6, sc-398103) and protein A/G beads were obtained from Santa Cruz Biotechnologies (Santa Cruz, CA, USA). Mouse monoclonal actin (clone C4) IgG (69100) was from MP Biomedicals LLC, Irvine, CA, USA. Donkey anti-goat IgG Alexa Fluor 647 was purchased from ThermoFisher Scientific (Waltham, MA, USA). Mouse monoclonal anti PSMA6 (α6) was a gift from Ariel Stanhill (Faculty of Medicine, Technion, Haifa, Israel). Horseradish peroxidase-conjugated bovine anti-goat IgG, goat anti-rabbit IgG, and goat anti-mouse IgG were obtained from Jackson ImmunoResearch Laboratories (West Grove, PA, USA).

### 4.3. Tumor Sample Processing

Brain samples from three transgenic mice of tumors originating from neuronal cells, astrocytes and neural stem cells (SC) [20] were gift of Dinorah Friedmann-Morvinski of Tel Aviv University. Human biopsies were obtained from individuals diagnosed and operated on at Ha’Emek Medical Center, Afula Israel. All patients were diagnosed with adenocarcinoma of the colon or rectum, with tumors that were graded at least B2 or higher. Biopsies were obtained during surgery upon written consent from patients according to the Helsinki regulations (Ha’Emek Medical Center- 0049–19). Samples were flash-frozen in liquid N_2_ and frozen pending analysis. For identification of COX-2 expression patterns, samples were placed on an ice-filled Petri dish, washed with ice-cold PBS and cut into small pieces. These pieces were then transferred into a test tube containing 2 mL ice-cold PBS supplemented with Complete Protease Inhibitor cocktail tablets (Sigma Aldrich, Saint Louis, MI, USA) and 0.1 mM PMSF and homogenized on ice using the Polytron tissue homogenizer. Remaining tissue debris was removed by filtration through a nylon mesh strainer (70 μm) and the homogenate was then centrifuged at 1000 × *g* for 10 min at 4 °C. The resulting pellet was resuspended in 0.5 mL RIPA/SDS (50 mM Tris pH = 8, 150 mM NaCl, 5 mM EDTA, 1% *v/v* NP-40, 0.5%, 0.5% *w/v* deoxycholic acid, 0.1% *w/v* SDS, 10 mM NaF, 0.1 mM PMSF and Complete Protease Inhibitor cocktail tablets with 0.1 mM PMSF and Complete Protease Inhibitor cocktail tablets and incubated on ice for 10 min. Samples were then centrifuged at 17,000 × *g* for 10 min at 4 °C, the supernatant was collected and protein levels were determined using the Bradford Assay (Bio Rad, Hercules, CA, USA). 100 μg of total tissue lysate was subject to SDS-PAGE.

### 4.4. Cell Culture and Transfection

Human embryonic kidney HEK 293 from the American Type Culture Collection repository (Manass, VA, USA), were cultured in DMEM, and supplemented with 10% heat-inactivated fetal bovine serum and 100 U/mL penicillin and streptomycin (Biological Industries, Beit HaEmek, Israel). Transient transfections were carried out in sub-confluent monolayers (70–80%) using Polyjet (SignaGen Laboratories, Rockville, MD, USA) at ratio of 1:3 (*w/w*) cDNA, according to the manufacturer’s instructions.

### 4.5. cDNA Constructs

pcDNA5/FRT/TO encoding human COX-2 was a gift from Prof. William L. Smith, University of Michigan. All other constructs were generated in the laboratory using the primers listed in Table 1. Cloning was verified at the core sequencing facilities of Hylabs (Rehovoth, Israel). K598A, K598I and K598E COX-2 primers were inserted into the pcDNA/FRT/TO vector between BamHI and HindIII restriction sites. Cloning of all other constructs was done using QuikChange Lightning Site-Directed Mutagenesis (Agilent Technologies, Santa Clara, CA, USA), according to the Manufacturer’s instructions.

### 4.6. Cell Extraction, Immunoprecipitation and Immunoblotting

Monolayers were washed twice with ice-cold PBS and lysed in RIPA/SDS buffer. Protein concentrations were determined with the Bradford Assay (Bio-Rad Laboratories, Hercules, CA, USA). Samples of total cell lysates were prepared in Laemmli sample buffer and 50–100 μg were used. In experiments of immunoprecipitation (IP), samples were centrifuged at 14,000 × *g* at 4 °C for 10 min, and the supernatants were collected, and protein levels were determined. Equal amounts of total protein were used for IP in the range of 0.5–0.8 mg total protein. Immunoprecipitations were performed using 2 μg of the appropriate primary antibody and 30 μL of a 50% slurry of protein A/G plus-agarose immunoreagent and agitated overnight at 4 °C. Immune complexes were washed three times with ice-cold RIPA/SDS, diluted in 30 μL of 2 × Laemmli sample buffer, and resolved by SDS-PAGE. Nitrocellulose membranes containing the immuno-complexes or total cell lysate proteins were incubated with primary antibodies at a dilution of 1:500–1000. Proteins were visualized by a WesternBright ECL (AdvanstaMenlo Park, CA, USA) and quantified using Amersham Imager 600 (GE, Buckinghamshire, UK) and quantified using Quantity One -1D analysis software.

Subcellular fractionation was done using the Rapid, Efficient And Practical (REAP) method [37]. Protocol was carried out exactly as described, except for the addition of Complete and 0.1 mM PMSF to the lysis buffer.

### 4.7. Flow Cytometry

Cells were washed twice with PBS and resuspended in 150–200 μL PBS for cytometric analysis. The samples were analyzed using BD FACSCanto II flow cytometer with DACSDiva software (BD Biosciences, San Jose, CA, USA). Gates were set to exclude necrotic cells and cellular debris and the fluorescence intensity of events within the gated regions was quantified. Data were collected from 10000 events for each sample. Apoptosis was measured using MEBCYTO-Apoptosis kit (Annexin V-FITC Kit) from Medical & Biological Laboratories (Nagano, Japan).

### 4.8. Microscopy

Cells were grown on 13-mm glass coverslips. Following transfection, cells were fixed with 4% paraformaldehyde, washed with PBS and blocked in PB buffer (1% BSA and 0.1% Triton X-100) for 5 min. Samples were then incubated with anti-COX-2 (1:200) for 1 h, washed three times with PBS, and incubated with Alexa Fluor 647 donkey anti-goat IgG (1:200) and Ki67 (1:200) for 1 h. Following three subsequent washes with PBS, samples were mounted onto glass slides using Mowiol (Sigma Aldrich, Saint Louis, MI, USA) and visualized using the laser scanning multiphoton confocal microscope A1 MP^+^ (Nikon, Melville NY, USA) at a 100 × magnification. All images were acquired using the same exposure conditions.

### 4.9. Proliferation assays

10^5^ HEK293 cells were cultured in 24-well plates. On the next day, cells were transfected with 0.5 μg of the various constructs and the effect on cell proliferation was assessed 24–48 h post-transfection using 3-bis-(2-methoxy-4-nitro-5-sulfophenyl)-(2*H*)-tetrazolium-5-carboxanilide (XTT) kit (Biological Industries, Beit HaEmek, Israel). Each experimental point contained 4–8 technical repeats and was performed in a minimum of three biological repeats.

For live cell tracking experiments, 3 × 10^4^ HEK 293 cells were seeded into 24-well dishes and transfected the next day as described above. Plates were placed in the IncuCyte^®^ ZOOM live-cell analysis system (Essen Bioscience, Ann Arbor, MI, USA) for 48 h and snapshots were taken every 30 min. Percent confluence was analyzed over time using the IncuCyte^®^ ZOOM Software at the Biomedical Core Facility, Rappaport Faculty of Medicine, Technion, Israel. Each experimental condition contained three repeats. Data of each condition was normalized to the value at time zero (first reading) to control for technical variability of the replicates.

### 4.10. COX-2 Activity Measurements

1.8 × 10^5^ HEK 293 cells were seeded in 12-well dishes and transfected as above. 16 h post-transfection, the media was syphoned, the cells were washed twice with warm PBS and incubated in 500 μL of serum-free DMEM containing 50 μM AA or vehicle for 30 min. At the end of the experiment, the supernatant was collected and the levels of PGE2 were analyzed using the Prostaglandin E_2_ Assay (R&D Systems, Minneapolis, MN, USA), according to the manufacturer’s instruction.

### 4.11. Transcriptome Analyses and Statistics

HEK 293 cells were transfected with either empty vector (Mock), WT COX-2 or K598R COX-2. Total RNA was prepared in three biological repeats using the Quick-RNA MiniPrep kit (Zymo Research, Irvine, CA, USA). Library preparation was performed using NEBNext Ultra RNA library Prep kit for Illumina (ThermoFischer Scientific, Waltham, MA USA), according to the Manufacturer’s protocol. Sequencing (single-read, 50bp) was carried out using the Illumina HiSeq 2500 at the TGC-Technion Genome center (Technion, Haifa, Israel). Sequence reads were aligned to the human reference genome version GRCh37 using Tophat (2.0.9). Gene expression levels were quantified using Htseq-count (0.6.1-py2.7) and differential expression was analyzed using EdgeR (3.2.4). Differential expression data were filtered based on log_2_FC ≥ 0.9, significance cutoff (Benjamini-Hochberg corrected *p*-value ≤ 0.05), and minimal reading levels (CPM ≥ 1). We performed GO enrichment analysis using DAVID Bioinformatics resources 6.8 [38,39]. Enrichment was considered significant for FDR adjusted *p*-value < 0.05. STRING Protein-Protein Interaction Networks Functional Enrichment Analysis was used to map the connections between genes and proteins that were common to transcriptomic and proteomic analyses. The highest degree of confidence prediction (0.9) was used [40].

### 4.12. Liquid Chromatography–Mass Spectrometry (LC-MS/MS)

Samples for proteomic analyses were ran on 1D-SD gel, following by staining with Coomassie brilliant blue dye (R-250 Dye, ThermoFisher Scientific, Waltham, MA, USA) for 1 h, and overnight destaining with double-distilled water. Samples were then analyzed by the Proteomics Services Platform (MASSPEC) at the Weizmann Institute of Science. Stained fragment bands were excised and underwent tryptic digest preparation. Twenty microliters of extracted tryptic peptides were analyzed using 4000 Q TRAP^®^ (MDS-Sciex, Concord, ON, Canada) liquid chromatography triple quadrupole/linear ion trap mass spectrometer. Peaklists were generated with Bioanalyst 1.4 (Mascot script) and submitted to Mascot (Matrix Science, Inc., Boston, MA) software for database search analysis against the UniProtKB non-redundant database.

### 4.13. Statistic and Software

All bars represent mean + SEM. Statistical analyses were done using the GraphPad Prism Software. Unless otherwise stated, statistical significance was determined by one-way ANOVA. Post-hoc analysis was performed with Tukey multi-comparison test when appropriate. *p*-values < 0.05 were considered significant.

## Figures and Tables

**Table 1:** Primers used for cloning.

Clone	Primers	Template
K598R	CAATCCCACAGTACTACTAAGAGAACGTTCGACTGAACTG	FL COX-2
	CAGTTCAGTCGAACGTTCTCTTAGTAGTACTGTGGGATTG	
K598A	ATTAAGCTTATGCTCGCCCGCGCCCTG	FL COX-2
	TGGATCCCTACAGTTCAGTCGAACGTTCGTGTAGTAGTACTGTGGGATTG	
K598I	ATTAAGCTTATGCTCGCCCGCGCCCTG	FL COX-2
	TGGATCCCTACAGTTCAGTCGAACGTTCTATTAGTAGTACTGTGGGATTG	
K598E	ATTAAGCTTATGCTCGCCCGCGCCCTG	FL COX-2
	TGGATCCCTACAGTTCAGTCGAACGTTCTTCTAGTAGTACTGTGGGATTG	
R106A K598A	GTAAGTTGGTGGACTGTCAATCACATGTGATGCGGAT	COX-2 K598A
	TTATGAGTTATGTGTTGACATCCGCATCACATGTGAT	
R106A L109V K598A	GTAAGTTGGTGGACTGTCAATCACATGTGATGCGGATGTCAACACATAACTCAT AA	COX-2 K598A
	TTATGAGTTATGTGTTGACATCCGCATCACATGTGATTGACAGTCCACCAACTTAC	
ΔNT	GTGAGAACCGTTTACCATGATTGACAGTCCACCAACTT	FL COX-2
	AAGTTGGTGGACTGTCAATCATGGTAAACGGTTCTCAC	
K598A G519A	ATATAACATTACCCATAAGTGCTTTCAAGGAGAATGGTGCTC	COX-2 K598A
	GAGCACCATTCTCCTTGAAAGCACTTATGGGTAATGTTATAT	

## Supplementary Materials

Supplementary materials can be found at https://www.mdpi.com/1422-0067/21/9/3195/s1.
