# Supplementary material for: Limited Proteolysis of Cyclooxygenase-2 Enhances Cell Proliferation
Source: Int J Mol Sci. 2020 Apr 30;21(9):3195. doi: 10.3390/ijms21093195 (PMC7246915; doi:10.3390/ijms21093195)
Supplement: Supplementary file 1 [file ijms-21-03195-s001.zip › Suppl. 1.docx]

**
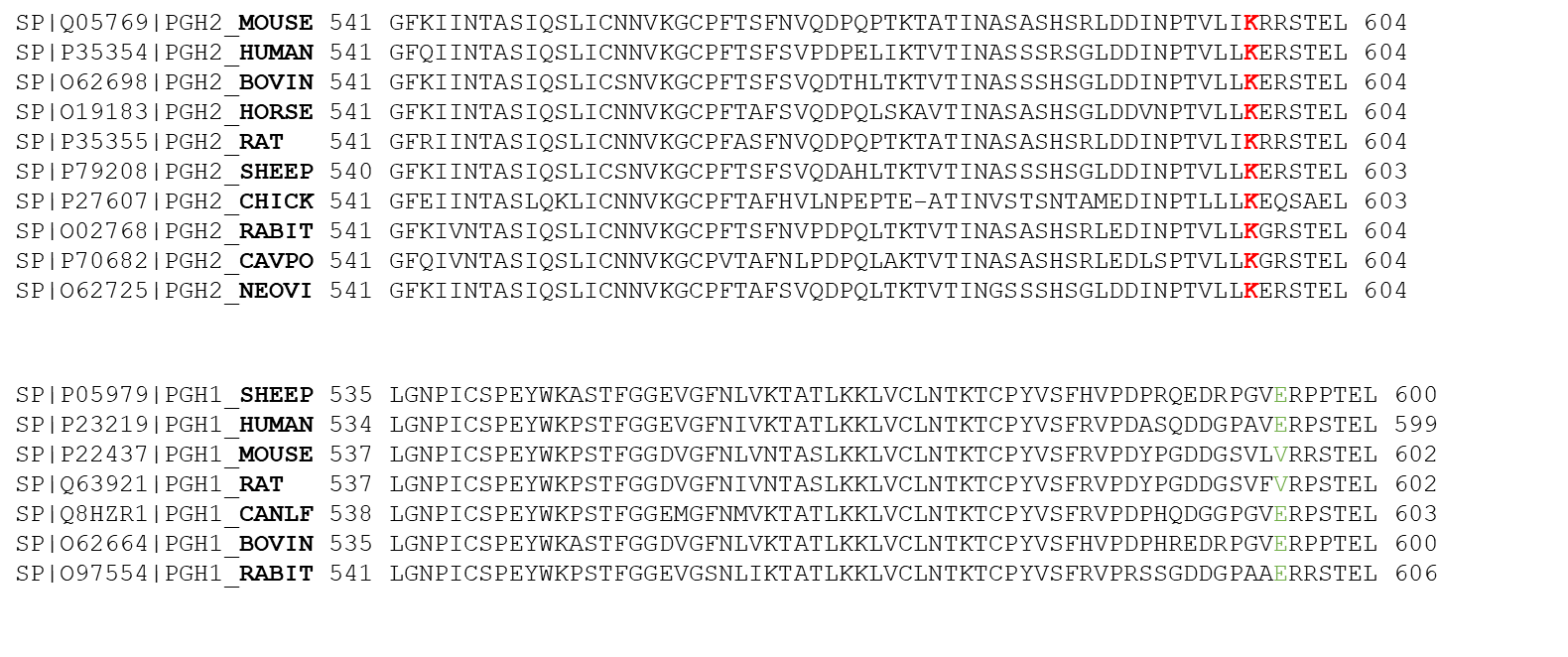
A**

**B**

**Supplementary Fig. 1: Multiple sequence alignment of the carboxyl tail of COX-2 (A) and COX-1 (B)**. Whereas the lysine residue in COX-2 is conserved in all sequenced vertebrates, this is not the case for COX-1, which shows the presence either glutamic acid or valine in the same position.
